# Associations of age and gender differences with career aspirations and burnout among Chinese undergraduate dental students

**DOI:** 10.3389/fmed.2026.1872239

**Published:** 2026-08-24

**Authors:** Zhe Chen, Ke Wang, Rongkai Cao, Chenyuan Sun, Min Zhang, Yujie Cao

**Affiliations:** 1 Department of Stomatology, the First Affiliated Hospital, Fujian Medical University, Fuzhou, China; 2 Department of Stomatology, National Regional Medical Center, Binhai Campus of the First Affiliated Hospital, Fujian Medical University, Fuzhou, China; 3 Department of Oromaxillofacial Head and Neck Surgery, Center for Oromaxillofacial Head and Neck Disorders, Huashan Hospital, Fudan University, Shanghai, China; 4 Shanghai Engineering Research Center of Tooth Restoration and Regeneration and Tongji Research Institute of Stomatology and Stomatological Hospital and Dental School, Tongji University, Shanghai, China; 5 Division of Applied Oral Sciences and Community Dental Care, Faculty of Dentistry, The University of Hong Kong, Hong Kong SAR, China

**Keywords:** age, career aspirations, diversity, gender, undergraduate dental students

## Abstract

**Introduction:**

This cross-sectional study sought to explore associations of age and gender with career aspirations and burnout symptoms among undergraduate dental students in China.

**Methods:**

An online survey was distributed to 240 subjects in December 2025, targeting current undergraduate dental students at Chinese universities. The survey outcomes included variables related to career choice and specialization plans. Additionally, the Burnout Clinical Subtype Questionnaire (BCSQ-12) was selected to estimate the burnout of the respondents. Chi-squared test was used to compare career aspirations across different age and gender groups. For burnout symptoms, an independent samples *t*-test was applied to compare burnout scores between male and female students and a one-way analysis of variance (ANOVA) was used to test score differences across three age strata.

**Results:**

Females were more likely to recognize male career advantages than males. Significant age differences were observed in intention to specialize in dentistry and influence of gender on career success. For the first specialty choice in dentistry, younger and female respondents are more likely to choose Orthodontics and Endodontics, while male and older subjects prefer Prosthodontics, Oral implantology, and Oral and maxillofacial surgery. Among the 240 subjects, the scores for overload, lack of development, and neglect were 10.75 ± 4.98, 11.04 ± 2.97, and 8.21 ± 3.86, respectively. Significant age-group differences were found in the overload and neglect of burnout, with older students scoring higher on these two domains, while no age variation was detected for lack of development. No statistically significant gender differences were identified across all three BCSQ-12 burnout dimensions.

**Discussion:**

Age correlates with career perceptions, specialization willingness and two burnout subscales, whereas gender only affects perceptions of gender-related career success with no significant burnout differences. By confirming these specific gender and age-related issues, corresponding strategies can be developed and implemented to enhance the environment of dental education.

## 1 Introduction

Chinese modern dental education started in the early 20th century, with over 100 dental schools in operation currently (1). Recent years have seen substantial revisions to Chinese dental education informed by educational experts, student feedback, and international dental curricula, with a special focus on the standardized residency training program (2). University dental students can now pursue various careers, including becoming a specialist dentist, general practitioner, educator, or civil servant (3). Among specialization options, orthodontics is the most popular, while Preventive dentistry and basal subjects face challenges in attracting students (3).

Undoubtedly, the most essential option for undergraduate dental students after graduation is choosing their careers. Research has shown that this choice is influenced by various factors, including flexible working hours, educational debt, gender, and the influence of parents' jobs (4). Understanding dental students' career choices can aid educators in better selecting and training them by adjusting curricula to enhance teaching content. Additionally, through a comprehensive study of future plans, students can develop more effective career strategies, and targeted measures can be implemented in the labor market to attract graduates.

Traditionally, burnout is defined as a three-dimensional syndrome comprising reduced personal accomplishment, emotional exhaustion, and depersonalization (5). Among students, burnout is marked by feelings of mental exhaustion and incompetence because of an inability to complete all required tasks (6). Research indicates that burnout syndrome is especially prevalent among university students in health majors such as medicine (7). Dentists, in particular, are highly susceptible to burnout because of the nature of their work in clinical settings (8). Additionally, both dental practice and education are well-documented sources of stress (9). Burnout is often most severe as dentists start their career. Consequently, dental schools are encouraged to integrate stress management training into the curricula (10).

Over the past several decades, the dental profession has undergone significant feminization worldwide. In many European countries, including Germany, Finland, and the UK, females make up more than 50% of dentists (11). Currently, notable differences exist in genders regarding motivational reasons for selecting dentistry. For instance, one research found significant differences between males and females in the desire to help others and in balancing work and family life (12). Moreover, gender differences in burnout exist as Qamar et al. found that emotional burnout was higher in female doctors compared to males (13). Consequently, it is important to analyze potential risks associated with the feminization of dentistry, as structural changes, such as an increased number of female practitioners, must be managed effectively, particularly in rapidly growing economies (14). In addition to gender, factors such as region, age, and race may also exert potential associations (15, 16).

Based on the evidence above, career aspirations and burnout levels are crucial for students, as these impact their future lives. However, little is known about these aspects among undergraduate dental students and their influencing factors. It is necessary to elucidate career aspirations and the prevalence of burnout, as well as potential associations related to age and gender, to inform improvement measures for dental students. Therefore, this study aimed to explore associations of age and gender with career aspirations and burnout symptoms among undergraduate dental students. The null hypothesis is that age and gender have no significant associations with dental students' career aspirations and burnout symptoms.

## 2 Methods

### 2.1 Study design

The present cross-sectional observational study was approved by the Institutional Review Board (Protocol code 2025-SR-[98]). The sample size estimation was done using G^*^Power software with the significance level set at 5% and the power of the test at 80%. Considering a 10% rate of incomplete or invalid questionnaires, the expected recruitment sample size was 214. An online survey was distributed via an online platform in December 2025 to collect data on career aspirations and burnout, targeting current dental students. Inclusion criteria included full-time undergraduate dental students enrolled in dental schools who voluntarily agreed to participate and fully completed the online questionnaire. Exclusion criteria covered postgraduate students, intern dentists, licensed dentists, and students who declined survey participation. Before commencing, participants were informed of its purpose and assured of confidentiality. All participants completed the questionnaires voluntarily and anonymously.

### 2.2 Measurements

#### 2.2.1 Demographic characteristics

The demographics part collected data on gender, age, race, region, and year of study. To further investigate the potential effects of family background on career aspirations, 4 additional items were developed, including parents' occupation, parents' education level, family income, and source of living expenses and tuition (17, 18).

#### 2.2.2 Career aspirations

Questions in this section were designed based on previous studies examining the career choices and specialization plans of dental students, with necessary adaptations to fit specific Chinese contexts (18–20). This section included questions on post-graduation paths, preferred career path, opinions on gender influences in success in dentistry and career success, work patterns, intentions to specialize, and first specialty choice in dentistry. Pilot testing were performed among 40 dental students to adjust ambiguous items, completed forward-backward translation and cultural adaptation suitable for Chinese dental undergraduates. The Cronbach's alpha coefficient was 0.72, and the KMO value reached 0.65.

#### 2.2.3 Burnout clinical subtype questionnaire

The BCSQ-12, introduced in 2011, is used to assess educational burnout among students in medical fields (21). This questionnaire comprises 12 items evenly distributed in three dimensions: lack of development, overload, and neglect. Participants respond using a 7-point Likert scale ranging from 1 (“completely disagree”) to 7 (“completely agree”). The BCSQ-12 demonstrates acceptable internal consistency for each dimension and adequate criterion validity (22). Reliability and validity tests for the Chinese BCSQ-12 scale were conducted among the 40 sampled dental undergraduates, with the Cronbach's alpha value of 0.75 and KMO value of 0.7, demonstrating acceptable internal consistency and favorable construct validity.

### 2.3 Statistical analysis

Data analysis was performed using SPSS 27.0 (IBM Corp., Armonk, NY, USA). Qualitative data were reported as percentages, while quantitative data were recorded as means and standard deviations. The Kolmogorov–Smirnov test was used to assess normality, and Levene's test to evaluate homogeneity of variances. As the data were normally distributed and met the assumption of equal variances, a one-way ANOVA was used to compare the mean values of lack of development, overload, and neglect by age, while an independent *t*-test examined potential differences in burnout between males and females. Chi-squared tests were conducted to compare career aspirations. Multivariable regression models were further constructed to separately examine the independent associations of age and gender with career aspirations and burnout. All regression models fully adjusted for predefined confounding variables, including race, region, year of study, parents' occupation, parents' education level, family income, and source of living expenses and tuition. A p-value of less than 0.05 was regarded as significant and was marked with an asterisk (^*^).

## 3 Results

### 3.1 Demographic information

A total of 252 individuals initially responded to the survey, and 240 participants (95.2%) satisfied all predefined criteria, while the remaining 12 respondents were excluded due to incomplete questionnaire submissions. The demographic data of the subjects is summarized in Table 1. Overall, 108 (45.0%) were male participants, with an average age of 21.0 ± 2.5 years old. Most subjects came from Fujian (*n* = 200, 83.3%) and identified as Han ethnicity (*n* = 226, 94.2%). 22 (9.2%) subjects had parents who were dentists, while 153 (63.7%) had parents with a high school degree or lower. The respondents included students from all grades, with the proportions in years 1, 2, 3, 4, and 5 being 81 (33.8%), 39 (16.3%), 40 (16.7%), 43 (17.9%), and 37 (15.4%), respectively. Regarding family income, most subjects were distributed among three categories: 12,000–50,000 RMB (*n* = 72, 30.0%), 50,000–100,000 RMB (*n* = 56, 23.3%), and 100,000–500,000 RMB (*n* = 62, 25.8%). The main source of living expenses and tuition was financial support from parents and family (*n* = 212, 88.3%).

**Table 1:** Demographic information of the respondents.

Variables	Category	Number (%)
Age	< 20	76 (31.7%)
	20–22	108 (45.0%)
	>22	56 (23.3%)
Gender	Male	108 (45.0%)
	Female	132 (55.0%)
Race	Han	226 (94.2%)
	Others	14 (5.8%)
Region	Shanghai	40 (16.7%)
	Fujian	200 (83.3%)
Year of study	One	81 (33.8%)
	Two	39 (16.3%)
	Three	40 (16.7%)
	Four	43 (17.9%)
	Five	37 (15.4%)
Parents' occupation	Dentist	22 (9.2%)
	Clinician	17 (7.1%)
	Other professions	201 (83.8%)
Parents' education level	bachelor's degree or above	87 (36.3%)
	high school and below	153 (63.7%)
Family income	< 12,000	40 (16.7%)
	12,000–50,000	72 (30.0%)
	50,000–100,000	56 (23.3%)
	100,000–500,000	62 (25.8%)
	>500,000	10 (4.2%)
Source of living expenses and tuition	Parents and family	212 (88.3%)
	Loan	13 (5.4%)
	Part-time job	5 (2.1%)
	Scholarship	6 (2.5%)
	Others	4 (1.7%)

### 3.2 Career aspirations

Table 2 presents the association between age, gender, and career aspirations among undergraduate dental students in China. A significantly higher proportion of female students (26.5%) agreed that males hold greater career advantages in dentistry, compared with male students (10.2%). Conversely, male respondents were far more likely to believe career success opportunities were equal for both genders (83.3% males vs. 70.5% females). Regarding the gender advantage for general career success, nearly 41% of female students recognized males' overall career advantages, which was markedly higher than the proportion among male students (16.7%). The proportion of students who thought career success was equal for all genders declined sharply with age: 72.4% (< 20 years), 75.0% (20–22 years), and only 37.5% (>22 years). In addition, younger students were far more likely to remain unsure about specialization plans: 38.2% (< 20 years) and 34.3% (20–22 years), whereas merely 10.7% of students aged above 22 were undecided. No significant differences were found regarding work patterns or career paths after graduation.

**Table 2:** The underlying association between age, gender and career aspirations among university dental students in China.

Item	Category	*N* (%)	Age group					Gender			
			<20	20–22	>22	χ^2^	*P* value	Male	Female	χ^2^	*P* value
Likely to succeed in dentistry	Same	183 (76.3%)	61 (80.3%)	84 (77.8%)	38 (67.9%)	5.78	0.22	90 (83.3%)	93 (70.5%)	11.10	< 0.01^*^
	Male	46 (19.2%)	14 (18.4%)	17 (15.7%)	15 (26.8%)			11 (10.2%)	35 (26.5%)		
	Female	11 (4.6%)	1 (1.3%)	7 (6.5%)	3 (5.4%)			7 (6.5%)	4 (3.0%)		
Advantage with regards to career success	Same	157 (65.4%)	55 (72.4%)	81 (75.0%)	21 (37.5%)	26.16	< 0.01^*^	85 (78.7%)	72 (54.5%)	16.94	< 0.01^*^
	Male	72 (30.0%)	19 (25.0%)	22 (20.4%)	31 (55.4%)			18 (16.7%)	54 (40.9%)		
	Female	11 (4.6%)	2 (2.6%)	5 (4.6%)	4 (7.1%)			5 (4.6%)	6 (4.5%)		
Intention to specialize in a specific area of dentistry	Yes	125 (52.1%)	37 (48.7%)	49 (45.4%)	39 (69.6%)	15.11	< 0.01^*^	60 (55.6%)	65 (49.2%)	1.08	0.58
	No	43 (17.9%)	10 (13.2%)	22 (20.4%)	11 (19.6%)			17 (15.7%)	26 (19.7%)		
	Unsure	72 (30.0%)	29 (38.2%)	37 (34.3%)	6 (10.7%)			31 (28.7%)	41 (31.1%)		
Plan of work patterns	Full-time	221 (92.1%)	70 (92.1%)	97 (89.8%)	54 (96.4%)	2.21	0.33	100 (92.6%)	121 (91.7%)	0.07	0.79
	Part-time	19 (7.9%)	6 (7.9%)	11 (10.2%)	2 (3.6%)			8 (7.4%)	11 (8.3%)		
Already thought about Path after graduation	Sure	72 (30.0%)	18 (23.7%)	31 (28.7%)	23 (41.1%)	5.86	0.21	37 (34.3%)	35 (26.5%)	4.59	0.10
	Tend to	150 (62.5%)	52 (68.4%)	67 (62.0%)	31 (55.4%)			60 (55.6%)	90 (68.2%)		
	Have not considered	18 (7.5%)	6 (7.9%)	10 (9.3%)	2 (3.6%)			11 (10.2%)	7 (5.3%)		

All percentages are column percentages calculated within each age/gender subgroup. ^*^P < 0.05 indicates statistically significant difference.

Table 3 displays multivariate logistic regression results adjusted for race, region, year of study, parents' occupation, parents' education level, family income, and source of living expenses and tuition. Gender exerted a significant association with the perception that males own greater advantages in achieving success in dentistry (Wald χ^2^ = 3.84, *p* = 0.04, OR = 0.25, 95% CI: 0.06–1.08). Students aged 20–22 years had significantly higher odds of holding the opinion that career success opportunities were equal for all genders when evaluating general career advantages (Wald χ^2^ = 3.90, *p* = 0.04, OR = 4.92, 95% CI: 1.01–23.93). Age group was strongly associated with the intention to pursue specialized dental training, with students aged < 20 (Wald χ^2^ = 15.50, *p* < 0.01, OR = 0.04, 95% CI: 0.01–0.19) and those aged 20–22 (Wald χ^2^ = 9.53, *p* < 0.01, OR = 0.13, 95% CI: 0.04–0.48) showing significantly lower odds of planning specialization relative to the reference group; meanwhile, students younger than 20 years (Wald χ^2^ = 14.75, *p* < 0.01, OR = 0.02, 95% CI: 0.01–0.16) and those aged 20–22 years (Wald χ^2^ = 3.83, *p* = 0.04, OR = 0.23, 95% CI: 0.05–1.02) also had markedly reduced odds of reporting no intention to specialize, while no significant correlations were found between age/gender and work patterns or postgraduate career planning certainty.

**Table 3:** Multivariate logistic regression of relationship between age, gender and career aspirations among university dental students in China.

Item	Category	Regression statistics	Age group			Gender	
			<20	20–22	>22	Male	Female
Likely to succeed in dentistry	Same	WaldX2	0.90	0.05		0.14	
		*p*	0.34	0.83		0.71	
		OR (95% CI)	2.74 (0.34, 22.15)	1.19 (0.24, 5.84)		0.78 (0.21, 2.84)	
	Male	WaldX2	0.01	0.29		3.84	
		*p*	0.93	0.59		0.04^*^	
		OR (95% CI)	1.10 (0.12, 10.27)	0.62 (0.11, 3.50)		0.25 (0.06, 1.08)	
	Female						
Advantage with regards to career success	Same	WaldX2	0.73	3.90		0.55	
		*p*	0.39	0.04^*^		0.46	
		OR (95% CI)	2.56 (0.30, 21.96)	4.92 (1.01, 23.93)		1.70 (0.42, 6.80)	
	Male	WaldX2	0.18	0.01		0.46	
		*p*	0.67	0.93		0.50	
		OR (95% CI)	0.63 (0.07, 5.52)	1.07 (0.22, 5.32)		0.61 (0.14, 2.56)	
	Female						
Intention to specialize in a specific area of dentistry	Yes	WaldX2	15.50	9.53		0.64	
		*p*	< 0.01^*^	< 0.01^*^		0.42	
		OR (95% CI)	0.04 (0.01, 0.19)	0.13 (0.04, 0.48)		1.30 (0.68, 2.50)	
	No	WaldX2	14.75	3.83		0.01	
		*p*	< 0.01^*^	0.04^*^		0.98	
		OR (95% CI)	0.02 (0.01, 0.16)	0.23 (0.05, 1.02)		1.01 (0.42, 2.44)	
	Unsure						
Plan of work patterns	Full-time	WaldX2	0.38	0.48		0.04	
		*p*	0.54	0.48		0.85	
		OR (95% CI)	0.50 (0.06, 4.49)	0.55 (0.10, 2.98)		1.11 (0.37, 3.32)	
	Part-time						
Already thought about Path after graduation	Sure	WaldX2	0.23	1.31		0.01	
		*p*	0.63	0.25		0.98	
		OR (95% CI)	0.57 (0.05, 5.92)	0.34 (0.05, 2.16)		0.98 (0.30, 3.26)	
	Tend to	WaldX2	0.02	1.14		1.70	
		*p*	0.89	0.29		0.19	
		OR (95% CI)	0.85 (0.09, 8.14)	0.38 (0.62, 2.28)		0.47 (0.15, 1.46)	
	Have not considered						

Analyses were adjusted for confounding variables: race, region, year of study, parents' occupation, parents' education level, family income, and source of living expenses and tuition. ^*^P < 0.05 indicates statistically significant difference.

**Figure 1:**
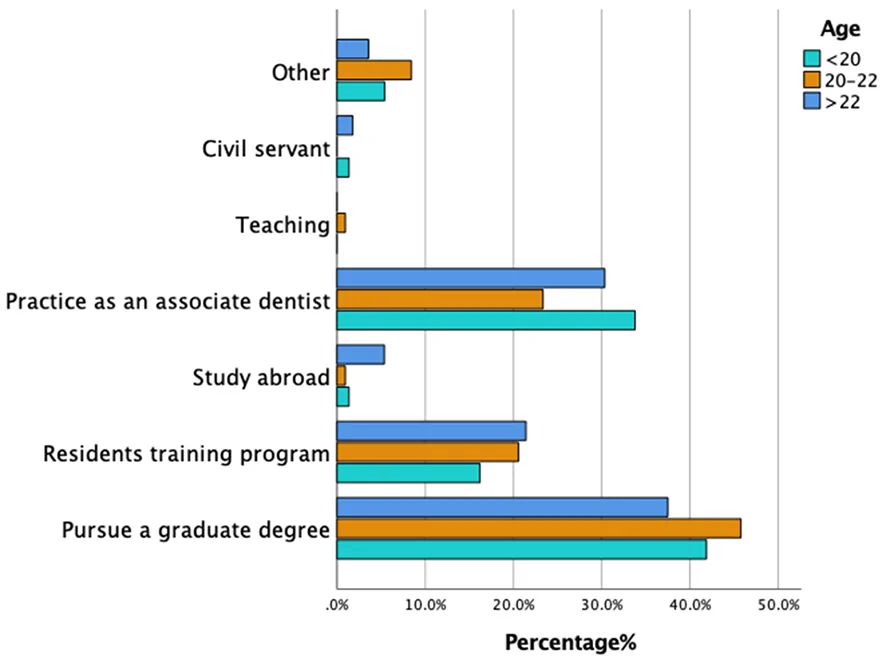
The preferred career path after graduation in different age groups.

**Figure 2:**
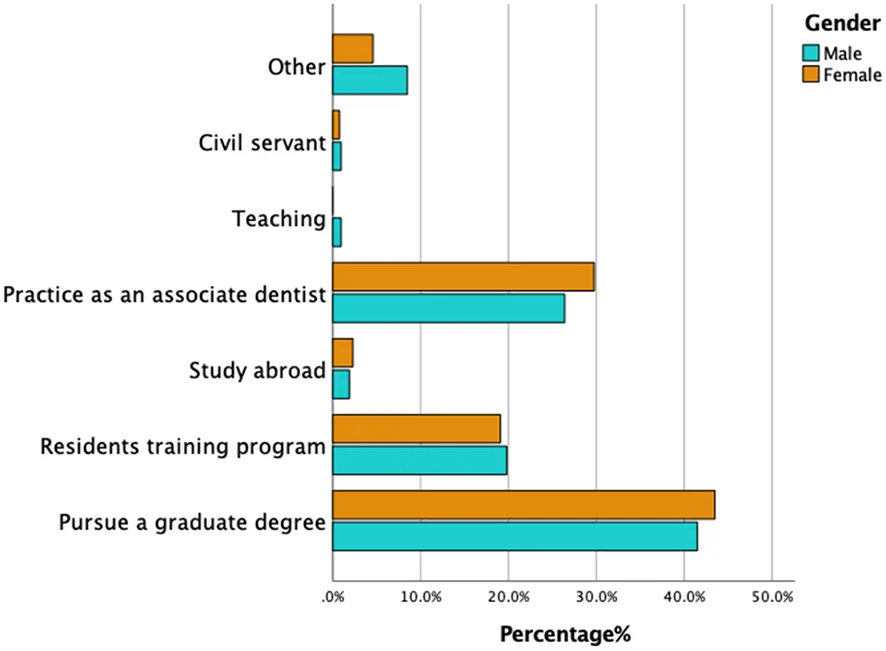
The preferred career path after graduation between males and females.

Figures 1, 2 illustrate the preferred career path of the subjects. Overall, pursuing a graduate degree remained the most popular postgraduate career choice across all age groups, including students younger than 20 years old. Among students under 20, though working as an associate dentist was a relatively more favored secondary option compared with older age cohorts, graduate study was still their primary preference. Students aged 20–22 showed the strongest willingness to pursue postgraduate education, while a small proportion of students older than 22 intended to study overseas (Figure 1). No significant differences were found between females and males regarding their preferred career path (Figure 2).

**Figure 3:**
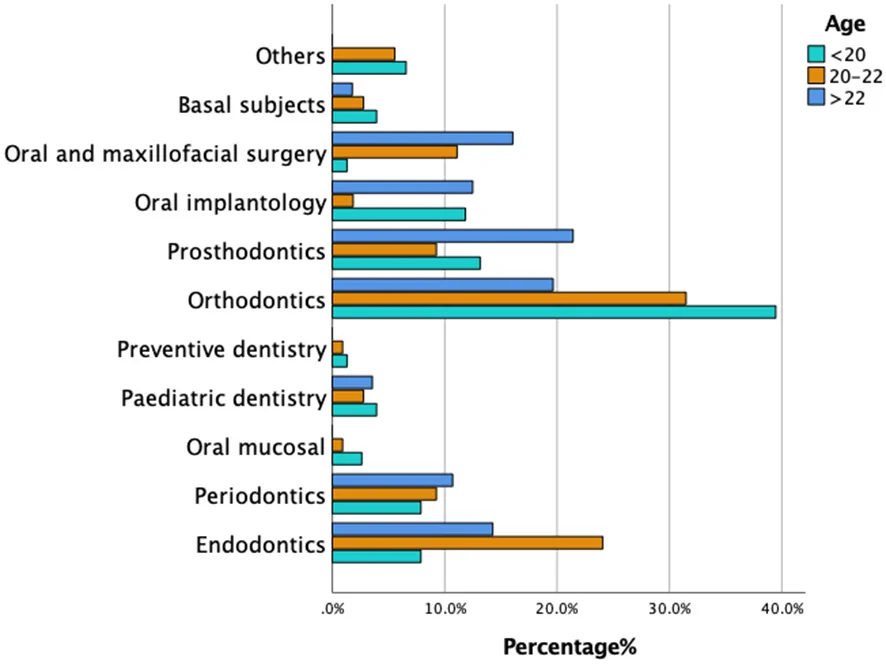
The first specialty choice in dentistry in different age groups.

**Figure 4:**
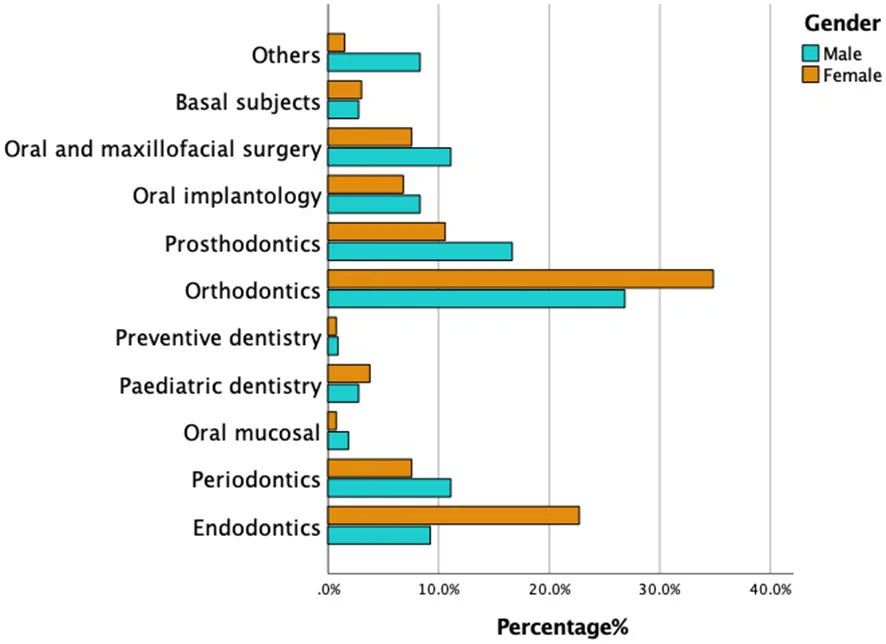
The first specialty choice in dentistry between males and females.

For first specialty choices in dentistry, Orthodontics was the most commonly favored dental specialty across all three age subgroups (< 20, 20–22, and >22 years) (Figure 3). When analyzing the age composition within individual specialties: participants who selected Endodontics were mainly concentrated in the 20–22 age group; Prosthodontics attracted a relatively larger share of students older than 22 years, compared with younger cohorts. Meanwhile, students aged >22 also exhibited higher interest in oral and maxillofacial surgery and oral implantology relative to participants under 20 years of age. Gender-based differences in specialty preference were prominent (Figure 4). Orthodontics was the most popular specialty among female students, with a far higher proportion of female respondents selecting orthodontics compared with male counterparts. Males showed greater preference for oral and maxillofacial surgery, Prosthodontics, oral implantology and Periodontology relative to females. For Endodontics, a larger share of female students expressed interest than males.

### 3.3 Burnout score

Table 4 summarizes the burnout scores across three dimensions and examines potential differences based on age and gender. The mean scores for overload, lack of development, and neglect were 10.75 ± 4.98, 11.04 ± 2.97, and 8.21 ± 3.86, respectively. ANOVA results indicated significant differences in overload and neglect among different age groups (*P* < 0.05). However, no significant differences were found between female and male respondents in burnout scores.

**Table 4:** The underlying association between age, gender and burnout (overload, lack of development and neglect) among university dental students in China.

Item		Overload	Lack of development	Neglect
Age	< 20	8.37 ± 3.76	10.61 ± 2.58	7.03 ± 3.59
	20–22	11.81 ± 5.07	11.23 ± 3.19	8.92 ± 4.00
	>22	11.93 ± 5.18	11.25 ± 3.04	8.46 ± 3.55
	*F* value	14.13	1.18	5.77
	*P* value	< 0.01^*^	0.31	< 0.01^*^
	Effect size	0.11 (0.04, 0.18)	0.01 (0.00, 0.04)	0.05 (0.01, 0.10)
Gender	Male	10.97 ± 5.40	10.84 ± 3.35	8.41 ± 4.10
	*F*emale	10.57 ± 4.62	11.20 ± 2.63	8.05 ± 3.63
	*t* value	0.63	−0.92	0.71
	*P* value	0.53	0.36	0.48
	Effect size	4.99 (−0.17, 0.34)	2.97 (−0.37, 0.14)	3.85 (−0.16, 0.35)

^*^P < 0.05 indicates statistically significant difference.

Table 5 shows covariate-adjusted multivariate linear regression results for burnout dimensions. Only age exhibited significant independent associations with overload (β = 0.23, *t* = 2.95, *p* < 0.01, 95% CI: 0.50, 2.51) and neglect (β = 0.41, *t* = 3.77, *p* < 0.01, 95% CI: 0.22, 1.39), indicating higher age was positively correlated with these two burnout domains. No significant correlations were found between age and lack of development, or between gender and any burnout subscale (all *p* > 0.05).

**Table 5:** Multivariate liner regression of relationship between age, gender and burnout (overload, lack of development and neglect) among university dental students in China.

Item		Overload	Lack of development	Neglect
Age	Beta	0.23	0.10	0.41
	*t* value	2.95	1.24	3.77
	*p* value	< 0.01^*^	0.21	< 0.01^*^
	95% CI	0.50, 2.51	−0.23, 1.01	0.22, 1.39
Gender	Beta	−0.04	0.07	−0.39
	*t* value	−0.64	1.09	−0.61
	*p* value	0.52	0.28	0.55
	95% CI	−1.64, 0.84	−0.34, 1.19	−1.29, 0.69

Analyses were adjusted for confounding variables: race, region, year of study, parents' occupation, parents' education level, family income, and source of living expenses and tuition. ^*^P < 0.05 indicates statistically significant difference.

## 4 Discussion

Diversity is a crucial factor in the advancement of human endeavors in science (23). Although it is challenging to provide an exhaustive definition of diversity, current research primarily considers dimensions such as gender, race, region, age, education, lifestyle, and cultural background (24). This study employed a self-administered questionnaire to investigate career aspirations and burnout among undergraduate dental students in China, along with the potential associations caused by demographic characteristics. Race was not considered as an influencing factor because the majority of respondents (95.4%) were of Han nationality, and the study's geographic limitation to Shanghai and Fujian impeded regional comparisons. Consequently, age and gender were selected as the main influencing parameters on career aspirations and burnout. For better comparison, respondents' ages were categorized into three groups on the basis of previous studies (21). The null hypothesis of the present study is rejected, as age exhibited significant independent associations with dental undergraduates' gendered career perceptions, sub-specialty training intentions, and two burnout sub-dimensions, while gender exerted a meaningful effect on students' judgement regarding gender-based career advantages.

Over the past several decades, diversity among university dental students has been a focus in Western countries where many institutions and programs have established commitments to sustaining diversity (25). Factors such as gender and race have been shown to influence the motivations and attitudes of university dental students (26). However, relatively little research has examined the diversity of undergraduate dental students in China and its potential impact on their career aspirations and mental health. According to data from the China Health Statistics Yearbook, the total number of dentists reached 278,000 in 2020, marking a 150.5% increase compared to 2010 (27). The diversity of the dental workforce has also undergone significant changes, including variations in age, gender, title, and educational background. For example, in 2010, the proportion of male and female dentists was 56.0% and 44.0%, respectively, while the percentage of female dentists rose to 52.6% in 2020. Participants in this study consist of 55% female dental students, which is consistent with the trend of changes in the occupational gender of dentists. By contrast, cross-national European research covering Germany, Finland and Turkey reported an even higher female proportion of 63% among dental students, demonstrating a more advanced feminization trend in Western dental education compared with the Chinese cohort in the present study (14). Dentists are the primary providers of oral health services, and dental students are the future dentists. By acknowledging gender and age-related issues, strategies can be developed to improve the dental education environment.

Significant differences emerged between male and female subjects regarding perceptions of success in dentistry and overall career success, with women more likely to perceive these advantages for men. Results of regression models indicates females were 75% less likely to hold the view that genders have equal career advantages in stomatology compared with males, demonstrating a moderate-to-large practical gender gap in career equity perception rather than only a minor statistical difference (OR = 0.25, 95% CI: 0.06–1.08, *p* = 0.04). This could be one possible explanation women in China often prioritize family over work, and the demanding workload and stress of dental studies can create conflicts between academic and professional commitments. Another explanation could be the impact of career breaks on women. Current professional training pathways are primarily designed around male dental professionals, posing challenges for women. Potential solutions include implementing part-time pathways or enhancing flexibility in training programs to facilitate advancement to senior positions. As women become increasingly active in the dental industry, addressing this disparity is crucial. Thus, measures are needed to maintain the diversity of dental practitioners and promote gender equality in dentistry.

Analysis of preferred career paths after graduation indicated that pursuing a graduate degree is the most popular choice among undergraduate dental students in China, followed by entering residency training programs and practicing as associate dentists, consistent with previous similar studies (18). Regarding specialty choices in dentistry, Orthodontics emerged as the most popular, particularly among female respondents. However, foundational subjects such as preventive dentistry and oral mucosal studies are currently less favored among dental students in China. This trend align with reports from both China and other countries, as orthodontics may potentially provide relatively higher remuneration (18, 28, 29). Therefore, administrators should emphasize developing strategies for less popular specialties to ensure diversity in professional choices.

The study results demonstrated no significant gender differences in burnout, aligning with previous research. For instance, a study by Galan et al. in Spain found no significant differences in educational burnout between female and male dental students (30). Similarly, research by Rajpurohit et al. using the Maslach Burnout Inventory-Student Survey also reported no significant gender differences among university dental students (31). These findings corroborate our results, suggesting that the gender gap in burnout among dental students has narrowed. Age presented moderate positive predictive effects on two burnout dimensions: overload (β = 0.23, 95% CI: 0.50–2.51, *p* < 0.01) and neglect (β = 0.41, 95% CI: 0.22–1.39, *p* < 0.01). The Beta value of 0.41 for neglect represents a stronger practical age effect than overload; every one-year increase in student age correlates with a clear rise in neglect burnout scores, with all CI entirely positive, ruling out random sampling fluctuation. Notably, the Spanish study conducted by Montero-Marin et al. observed non-significant age-related disparities across all three burnout subdimensions among local dental students, which stands in direct contrast to the prominent age gradients of overload and neglect burnout identified within the Chinese sample in the current research (21). We speculate that this association may arise from senior students' need to balance theoretical coursework and clinical internships. Therefore, education departments and university administrators should consider curriculum adjustments for senior students to effectively integrate work and rest. Additionally, strengthening stress management training is crucial for enhancing the prevention of potential burnout among undergraduate students. There should be an emphasis on early screening for psychological issues in dental students, with increased attention to those who may face challenges, thereby providing timely support and solutions.

This study has several limitations. Firstly, data were primarily collected from universities in Shanghai and Fujian, which may lead to selection bias and restrict the generalizability of the findings. Besides, the existence of non-response bias cannot be ruled out, as only students who were willing to complete the questionnaire were included. All outcome indicators were measured via self-reported scales, which might be subject to reporting bias due to subjective perception. Additionally, although the mature BCSQ-12 was used, the validity and reliability of the entire questionnaire were not assessed in this study. Future research should consider conducting a larger-scale survey to validate these findings and provide a basis for formulating future strategies.

## 5 Conclusion

This study employed an online questionnaire to investigate the career aspirations and levels of burnout among undergraduate dental students in China. Specifically, age was significantly associated with students' perceptions of gender advantages in dental career success and their willingness to pursue specialized dental fields, while gender was linked to self-evaluated likelihood of career success. In terms of burnout, age showed significant associations with the overload and neglect dimensions of burnout, whereas no significant association was observed between gender and burnout. By addressing these gender and age-related issues, targeted strategies can be developed and implemented to enhance the dental education environment, thereby optimizing resource allocation for dentists and fostering a diverse range of dental practitioners in China.

## Data Availability

The raw data supporting the conclusions of this article will be made available by the authors, without undue reservation.
